# Childhood school segregation and later life sense of control and physical performance in the African American Health cohort

**DOI:** 10.1186/1471-2458-12-827

**Published:** 2012-09-27

**Authors:** Fredric D Wolinsky, Elena M Andresen, Theodore K Malmstrom, J Philip Miller, Mario Schootman, Douglas K Miller

**Affiliations:** 1 Department of Health Management and Policy, College of Public Health, the University of Iowa, Iowa City, IA, USA; 2 Department of Internal Medicine, Carver College of Medicine, the University of Iowa, Iowa City, IA, USA; 3 Department of Adult Nursing, College of Nursing, the University of Iowa, Iowa City, IA, USA; 4 Institute on Development and Disability, Oregon Health & Science University, Portland, OR, USA; 5 Department of Psychiatry and Neurology, Saint Louis University, St. Louis, MO, USA; 6 Department of Biostatistics, Washington University, St. Louis, MO, USA; 7 Department of Internal Medicine, Washington University, St. Louis, MO, USA; 8 Regenstrief Institute, Incorporated, Indianapolis, IN, USA; 9 Department of Internal Medicine, Indiana University, Indianapolis, IN, USA

## Abstract

**Background:**

The association between childhood school desegregation and later life sense of control and physical performance among African Americans is not clear. We hypothesized that childhood school desegregation adversely affected the sense of control of in later life, and that this reduced sense of control accounts in part for reduced physical performance.

**Methods:**

In-home follow-up assessments were completed in 2010 with 582 of the 58–74 year old men and women participating in the on-going African American Health cohort. We used these data to examine the relationship between (a) retrospective self-reports of attending segregated schools during one’s 1^st^-to-12^th^ grade education and one’s current sense of control, as well as (b) the association between current sense of control and physical performance. Multiple linear regression analysis with propensity score re-weighting was used.

**Results:**

Attending segregated schools for at least half of one’s 1^st^-to-12^th^ grade education was significantly associated with higher scores on the sense of control. Adjusting for all covariates and potential confounders, those receiving half or more of their 1^st^-to-12^th^ grade education in segregated schools had sense of control scores that were .886 points higher (*p* ≤ .01; standardized effect size = .22). Sense of control scores were independently (all *p* < .01) associated with better systolic blood pressure, grip strength, peak expiratory flow, chair stands, balance tests, and the Short Portable Physical Battery even after adjusting for all covariates and potential confounders. Moreover, sense of control scores either partially or fully mediated the statistically significant beneficial associations between childhood school segregation and physical performance.

**Conclusions:**

Childhood school desegregation was adversely associated with the sense of control of African Americans in later life, and this reduced sense of control appears, in part, to account for their poorer physical performance. The etiologic mechanism through which childhood school segregation at the time that this cohort experienced it improved the sense of control in later life, which subsequently led to better physical performance, has not been identified. We suspect, however, that the pathway involves greater exposure to racial solidarity, same-race students as peer role models and same-race teachers and principals as authority role models, the reduced likelihood of exposure to race-based discrimination or antagonism during their formative early lives, and greater exposure to encouragement and support for academic and life success.

## Background

Personal control [1-5], sometimes referred to as locus of control [6], sense of control [7,8], sense of coherence [9], mastery [10], or self-efficacy [11] has played an important role in the study of health and health behaviour [12-17], because:

“…compared to people who feel powerless to control their lives, people with a sense of control know more about health, they are more likely to initiate preventive behaviours like quitting smoking, exercising, or maintaining normal weight, and in consequence, they have better self-rated health, fewer illnesses, and lower rates of mortality” [15; p. 66].

As a result, personal control among older adults is viewed as a marker of successful aging, and further study of this construct is warranted [14,18].

A frequently observed association with personal control involves race [15]. African Americans generally report lower levels of personal control than non-Hispanic Whites or other minorities [15,19,20]. In most studies, however, race is simply used as a covariate, with primary attention focused on the effects of age, education, and health. Indeed, only two studies [20,21] have modelled personal control separately for older non-Hispanic Whites and African Americans. Thus, much remains to be known racial differences in personal control, especially since the dominant view is that personal control becomes relatively stable by early adulthood, with declines commencing after age 50 due to the progressively less favourable ratio of gains to losses in later life [9,13,14,22,23]. Therefore, any social processes that adversely affected school-aged African American children may have played an important role in the development of their personal control.

Along those lines, Buck [24] recently argued that desegregation left an ironic legacy for African Americans. He claimed that desegregation:

“…undermined one of the traditional centres of the black community: the school. In the segregated schools, black children had consistently seen other blacks succeeding in the academic world. The authority figures and role models—that is, the teachers and principals—were virtually always black. And the best students in black schools were black as well. That ended with desegregation” [24; pp. 3–4].

Based on Buck’s argument, we hypothesized that the sense of control among African Americans adults who attended segregated schools as children would be higher than that among African American adults who attended desegregated schools. Moreover, given the extensive literature linking the sense of control with better health outcomes, we hypothesized that the higher sense of control among African Americans adults who attended segregated schools as children would be independently associated with better physical performance in later life. Furthermore, we hypothesized that these effects were independent of potential confounders such as those risk factors and covariates traditionally found to be associated with the sense of control (e.g., demographic factors, socioeconomic status, and self-rated health), as well as regional differences in where the childhood schooling occurred, and current racial attitudes and beliefs, and resilience. Finally, we hypothesized that the association between childhood school segregation and physical performance in later life would be partially or fully mediated by the sense of control. Note that while our hypotheses and our evaluation of them flow directly from Buck’s argument, they do not address the etiologic pathways that he postulated.

Essentially, the etiological mechanism that Buck [24] proposed was that desegregation-induced erosions of traditional “black community” schools may have reduced or eliminated three protective effects for the children who attended them—the crystallization of racial solidarity, the advantage of having same-race students as role models and same-race teachers and principals as authority role models, and not having been discriminated against or antagonized at school on the basis of race [25,26]. Racial solidarity would have arisen from the collective identification associated with segregated schooling where all of one’s peer models, including the best students, star athletes, and most popular individuals were African American. African American students in segregated schools would also have had more exposure to role models of successful achievement reflected by their African American teachers and principals. And by attending segregated schools, African American students would not have been discriminated against or antagonized at school due to their race. Moreover, they would have been less likely to be discouraged due to any academic failures, and they would be more likely to have received greater levels of support encouraging their success. All of these are well-documented benefits for African Americans attending Historically Black Colleges and Universities (HBCUs) [27-29].

Buck’s [24] argument also resonates with Allport’s contact hypothesis [30] for optimizing intergroup relations, which provides a conceptual backdrop for understanding why U.S. school desegregation did not equate with integration [31,32]. That is, subsequent to the 1954 *Brown v Board ofEducation* U.S. Supreme Court ruling, a cascade of federal policies were implemented that were designed to better integrate African Americans into American society. Essentially, those policies assumed that desegregation would provide equal access to quality education, which had long been considered the springboard for achieving the American dream. As Pettigrew [33] noted, however, for desegregated schooling to achieve these goals, the four conditions necessary for the contact hypothesis—“equal group status within the situation, common goals, intergroup cooperation, and authority support”—were not met. Thus, while schools were legally desegregated, African American students were not functionally integrated into their new schools, and not all of the benefits that those schools should have provided African American students were received.

Although a growing number of studies have considered the effects of school segregation on health outcomes for children and young adults [34-38], no studies have focused on this association among older adults. Moreover, in a review of 39 studies on the association between segregation and health outcomes, 38 were found to have relied solely on measures of residential (rather than school) segregation [39], with the only study using school segregation having combined it into a composite with neighbourhood and church segregation [40]. Nonetheless, several studies have reported protective effects of living in clustered African American neighbourhoods on health outcomes [41]. Thus, the hypotheses for this study were consistent with prior reports, although our hypotheses focused on the independent relationship between school segregation in childhood (as a marker of exposure to the rich benefits of the “black community” school) and the sense of control in later life, and the sense of control in later life and physical performance with these constructs being measured at the individual rather than the community level. Measuring school segregation at the individual level enhances the validity of correct exposure classification by focusing on whether each study participant actually attended segregated schools rather than merely lived in an area where segregated schools were available within the community. Moreover, school segregation and residential segregation are rather different phenomena that would not necessarily have the same effects [39-41].

## Methods

### Cohort selection and sample

We investigated our hypotheses using data on the African American Health (AAH) cohort. The original AAH cohort included 998 African American men and women born in 1936–1950 whose baseline interviews occurred in 2000–2001 [42,43]. Participants lived in a poor inner-city area of St. Louis, Missouri, or the near northwest suburbs. They have been shown to be more socioeconomically and health disadvantaged than their counterparts in nationally representative studies [44]. Equal numbers of participants were randomly recruited from both areas, but because the inner city had fewer eligible persons, the probability of selection was higher there. Thus, sampling weights were used to adjust for the unequal selection probabilities. Inclusion criteria were self-reported Black or African American race and Mini-Mental Status Exam (MMSE) [45] scores ≥ 16 (which reflects an established cognitive status threshold for obtaining reliable and valid self-reports). A 76% response rate was obtained for the baseline in-home evaluations. Interim follow-ups were conducted at 1, 2, 3, 4, and 7 years. All data reported here come from the 9-year follow-up in-home evaluations that were conducted in 2010. Of the 998 original participants, 582 (58%) were re-interviewed, representing a 9-year retention rate of 67.8% among known survivors.

The AAH is a particularly well-suited data source with which to investigate our hypotheses for several reasons. First, although two-thirds of the AAH participants received the bulk of their 1^st^-to-12^th^ grade educations in Missouri, 28.6% completed most of their education in the South, and 5.2% completed their education mostly elsewhere. Thus, although all of the AAH participants resided in St. Louis at the onset of the study, there was sufficient regional variation in where their childhood education was obtained to support an empirical evaluation of regional schooling effects. Second, AAH participants were 3–19 years old when the 1954 *Brown v Board ofEducation* U.S. Supreme Court ruling put an end to *de jure* school segregation, which was well-established not just in Southern States or Border States like Kansas and Missouri, but throughout the U.S. at that time [46]. Third, AAH participants were 11–27 years old in 1962 when President Kennedy issued *Executive Order 11063* requiring federal agencies to “take all necessary and appropriate action to prevent segregation,” which was the first explicit federal policy statement against residential segregation, which is well-known to be the underlying cause of widespread *de facto* school segregation [47]. Fourth, AAH participants were 13–29 years old when Title IV of *The Civil Rights Actof 1964* authorized the federal government to file school desegregation cases. Finally, many states continue to have extensive *de facto* segregated schooling. For example, 40% of African American students in Missouri attend schools that are nearly (90% or more) all African American [48]. Thus, the AAH cohort experienced their 1^st^-to-12^th^ grade education either entirely or in some admixture of desegregated vs. segregated schools in Missouri, the South, or elsewhere, and their children and grandchildren continue to experience substantial *de facto* segregation.

### Potential selection bias

Because 416 of the 998 original participants were not re-interviewed, selection bias of the 2010 data relative to the original 2000–2001 AAH cohort is possible. To adjust for this, propensity score re-weighting methods were used [49-52]. This involved estimating a multivariable logistic regression model of whether or not each of the 998 original AAH participants was re-interviewed in 2010. The fit of that model was acceptable (*AUC* = .682) [53] and there was no evidence of heteroscedastic error (Hosmer-Lemeshow *p* value = .702 ) [54], indicating that observable selection bias could be addressed using this approach. The inverse of average participation rates within predicted probability quintiles were then used for re-weighting the original sampling weights, with rescaling of the weighted sample size to the actual number of participants interviewed in 2010. All results reported here reflect the propensity score re-weighted data, although analyses that relied on the original sampling weights reached the same conclusions.

### Measurement

#### The sense of control

Mirowsky’s 8-item index [7], developed in a nationally representative sample that included an oversample of African Americans, was used to measure the sense of control. Four statements (“I am responsible for my own successes”, “I can do just about anything I really set my mind to”, “my misfortunes are the result of mistakes I have made”, and “I am responsible for my failures”) reflect claiming control over both good and bad outcomes and are coded - 2, - 1, 0, 1, and 2 for strongly disagree, disagree, don't know, agree, and strongly agree. Four statements (“the really good things that happen to me are mostly luck”, “there's no sense planning a lot — if something good is going to happen, it will”, “most of my problems are due to bad breaks”, and, “I have little control over the bad things that happen to me”) reflect denying control over both good and bad outcomes and are reverse-coded.

Balancing claiming vs. denying control statements for good and for bad events cancels the agreement bias that commonly exists when using Likert-scaled items and improves the validity of the sense of control measure. For both non-Hispanic Whites and African Americans, internal consistency and test-retest reliability are good [13,55,56], and confirmatory factor analysis validation is robust [57]. In our main analyses, we used the overall sense of control score which is the sum of the responses to the 8 items, and ranges from maximally denying (−16) to maximally claiming control (+16). In sensitivity analyses to better characterize any observed association between childhood school segregation and the overall sense of control score, we also separately examined its two main components—the sum of the responses to the four items reflecting claiming control, and the sum of the responses to the four items reflecting denying control.

#### Childhood school segregation

Childhood school segregation was based on retrospective self-reported perceptions about attending segregated schools in response to two questions. Participants were first asked “Did you attend any racially segregated schools during the first through twelfth grades?” Participants responding “yes” were then asked how many years and for which particular grades this occurred. The percentage of one’s 1^st^-to-12^th^ grade education that occurred in segregated schools was calculated by normalizing the reported number of years of segregated schooling to the total reported number of years of schooling. For example, if an AAH participant reported having 10 years of schooling with 8 of these being in segregated schools, then that individual’s percentage of segregated schooling would be 80%. If, however, an AAH participant reported having 18 years of schooling with 8 of these being in segregated schools, then that individual’s percentage of segregated schooling would be 67%. Based on the observed distribution, these percentages were transformed into two dummy variables reflecting having been in segregated schools for 50% or more of one’s 1^st^-to-12^th^ grade education, or for 1-49%, vs. none (the reference category).

We readily acknowledge that this measure of childhood school segregation reflects retrospective self-perceptions of segregated schooling and the perceived extent of that exposure. As such it has limitations. It is not an objective, independent assessment of school segregation, nor can it be because information on the particular schools that each AAH participant attended was not obtained. This measure of childhood school segregation also does not differentiate between *de jure* vs. *de facto* segregation. Based on the widespread residential segregation in the 1930s-1960s [47] and the prevailing neighbourhood schools approach [57], *de facto* segregated schooling was common, while *de jure* segregation was less common and concentrated primarily in the Southern States and in the Border States. These limitations, however, bias the results toward the null, suggesting that any observed associations are likely to have been underestimated.

#### Region of schooling

While the dominant approach to education at the time was based on neighbourhood schooling [57], regional variations in school quality were known to exist. In particular, the quality of schooling, especially for African Americans, in the South has been widely viewed as having lower quality. Therefore, to separate regional from segregated schooling effects, two questions were asked of AAH participants. One was “In what State or States did you go to grade school,” while the other targeted “high school”. Participants were allowed to specify up to five States, and based on the observed response patterns two dummy variables were created. These reflect receiving most of one’s 1^st^-to-12^th^ grade education in the South (Alabama, Arkansas, Florida, Georgia, Kentucky, Louisiana, Mississippi, North Carolina, Oklahoma, South Carolina, Tennessee, Texas, and Virginia, based on the U.S. Census classification system) vs. in Missouri (the reference group) vs. elsewhere.

#### Traditional covariates and potential confounders

Our hypotheses specify (a) *independent* associations between childhood school segregation and the sense of control in later life, (b) *independent* associations between the sense of control and physical performance in later life, and (c) that the sense of control *partially or fullymediates* the beneficial associations between childhood school segregation and physical performance in later life. Therefore, it is essential that traditional predictors of the sense of control be included as covariates, and that potential confounders (such as racial attitudes and beliefs, and resilience) be included as well, even at the risk of over-adjustment. While including all these covariates and potential confounders might result in over-adjustment, this is a conservative error that results in the most rigorous evaluation of whether childhood school segregation is *independently* associated with the sense of control in later life, and whether the sense of control in later life is *independently* associated with physical performance.

At a minimum, there is general agreement that analyses examining the sense of control should include age, gender, marital status, educational attainment, employment status, income, and health, because these are known to be among the principal drivers of the sense of control [13,19,58]. Age was measured in years. Gender was coded as a binary contrast for men vs. women. Marital status was measured using a set of dummy variables reflecting never being married, being widowed, or being divorced or separated vs. being married (reference group). To minimize multicollinearity with the childhood segregated schooling indicators, educational attainment was used in the analysis as an ordered trichotomy reflecting (1) grade school, (2) high school, and (3) college. Employment status was measured using dummy variables for working for pay or being disabled vs. being retired (reference category). Income was measured using an unfolding bracketing approach, and given the observed distribution income was categorized as a binary indicator for household incomes of $25,000 or less vs. more. The traditional self-rated health question (Would you say your health is excellent, very good, good, fair, or poor?) was used to measure perceived health, and was coded from 1–5 for poor to excellent responses.

#### Racial attitudes and beliefs

Racial consciousness was measured using one item from the U.S. Behavioural Risk Factor Surveillance System [59]. It asked “How often do you think about your race? Would you say never, once a year, once a month, once a week, every day, every hour, or constantly?” Based on the response distribution, a binary indicator contrasting responses of “constantly” with all others was constructed.

To tap participants’ beliefs of being responsible for the well-being of the African American community, they were asked whether they strongly disagreed, disagreed, agreed, or strongly agreed (coded 1–4) with the statement: “It is my responsibility to help move the Black community forward.” Perceived discrimination was measured using Williams’ scale [60] (alpha = 0.82) asking participants to indicate how often (1 = never, 2 = rarely, 3 = sometimes, 4 = often) in their daily life nine things had happened to them. The nine things were (a) being treated with less courtesy or (b) respect than other people, (c) receiving poorer service in restaurants or (d) stores, (e) having people act as if you are not smart, (f) better than, or (g) afraid of you, (h) having people think you are dishonest, or (i) having people call you names. Scale scores ranged from 9 (no perceived discrimination) to 36 (maximal perceived discrimination).

#### Resilience

To parse out the potential overlap between resilience and the sense of control, the 10-item version [61] of the Conner-Davidson [62] resilience scale that was developed and validated in large samples of non-Hispanic White and African American participants (alpha = 0.88) was used. Participants responded to ten statements as (1) not true at all, (2) rarely true, (3) sometimes true, (4) often true, or (5) true nearly all of the time. The ten statements reflected the ability to adapt to change, to deal with whatever comes, to see the humorous side of problems, to recognize that coping with stress can strengthen me, to bounce back after an illness or hardship, achieve goals despite obstacles, stay focused under pressure, not be easily discouraged by failure, handle unpleasant feelings, and to think of myself as a strong person. The scale score ranged from minimal (10) to maximal (50) resilience.

#### Physical performance assessments

Although a number of self-reported measures of physical abilities are available in the AAH (indeed, in predicting the sense of control we adjusted for perceived health using the traditional self-rated health question), we focused on physical performance assessments. The reason for this was that the association between the sense of control and self-reported measures of physical abilities would be viewed as “weaker” evidence of the underlying relationship because both are self-reports and perceptions. Therefore, associations between the sense of control and physical performance assessments using standard epidemiologic field methods and well-established data collection protocols represent “stronger” evidence that is less likely to result from methodological artefacts.

Eight standard physical performance tests used in the AAH from its inception, all of which have demonstrated test-retest reliability as well as validity [63], were selected—systolic and diastolic blood pressure, grip strength, peak expiratory flow, gait speed, chair stands, balance tests, and the Short Portable Physical Battery (SPPB) [64,65]. Systolic and diastolic blood pressures (mm/hg) were the average of two trials spaced five minutes apart using electronic sphygmomanometers with participants seated in chair with both feet on the ground, and their left arm properly supported. After three sub-maximal familiarization trials, grip strength (kg) was the average of three trials using a handhold dynamometer while seated with both feet on the floor and the forearm and upper arm at a 90 degree angle. Peak expiratory flow (ml) was the average of three trials using a standard handheld flow meter. Gait speed (m/s) was the average of two trials using either a 3- or 4-meter course (based on interior space and clutter constraints in the participants’ homes), and was categorized as 0 for unable to perform, and 1–4 for the approximate quartiles of those performing the test [42]. Chair stands (with arms crossed over the chest) were measured in seconds from the initial sitting position to the top of the fifth rise, and were categorized as 0 for unable to perform and 1–4 for the approximate quartiles of those performing the test [42]. Balance was measured in the number of seconds (up to a maximum of 30) that the side-by-side, semi-tandem, and tandem stands were held, and was categorized as 0 for unable to perform any of these tests, and 1–4 for the approximate quartiles of those performing the test using the scoring method of Guralnik et al. [64,65]. The SPPB was the sum of the categorized scores on the gait speed, chair stands, and balance tests, and ranges from 0 (unable to perform any of these three tests) to a maximum of 12, with lower scores (especially those below 9) indicative of increased risk of future mobility impairment, poorer prognosis and recovery from newly diagnosed disease, and death [64-69].

### Analysis

Linear regression was used to examine the crude association of childhood school segregation with the sense of control in later life. Each covariate block was then serially introduced, adjusting for the region where one’s 1^st^-to-12^th^ grade education occurred, the traditional covariates and potential confounders, racial attitudes and beliefs, and resilience. Linear regression with the same serial decomposition approach was also used to examine the crude and adjusted associations of the sense of control in later life with physical performance. Finally, to evaluate whether the sense of control in adult life partially or fully mediated the crude association between childhood school segregation and physical performance in later life, linear regression was again used to examine the crude association, and whether it was altered by adjustment for the sense of control.

### Human subject approvals

The research reported here was supported by a U.S. National Institutes of Health (NIH) grant (R01 AG010436) to Dr Miller. The human subject protocol for this U.S. NIH grant was fully approved by Institutional Review Boards (IRBs) at Saint Louis University, the University of Iowa, Indiana University, Washington University, and the Oregon Health & Science University. Written informed consent was obtained from all participants at baseline, and at the 9-year follow-up interviews from which all of the data used here were taken.

## Results

### Descriptive data

Table 1 contains the percentages or means and standard deviations for all of the independent and dependent variables among the 582 AAH participants at the time of their 2010 in-home assessments. In brief, the average age was 65.8, 42.8% were men, 28.1% were divorced or separated, and about half (49.8%) had a high school education (mean = 11.4 years) with most of the rest (44.9%) having attended college (mean = 14.7 years). About one-third (30.3%) were working, 42.6% had annual incomes of $25,000 or less, and the average self-rated health was good. One-third (36.4%) thought about their race constantly, 79.6% felt responsible for the African American community, perceived discrimination was modest (24.7), and resilience was high (40.6). Segregated schools were attended during at least half of one’s 1^st^-to-12^th^ grade education by 40.2%, and about half (43.9%) of those with segregated schooling had that exposure all the time.

**Table 1:** **Percentages or means and standard deviations of the variables at the 9-year follow-up of the African American Health cohort in 2010 (N = 582 unless otherwise noted)**^**1**^

**Variable**	**Percentage**	**Mean**	**Standard Deviation**
*Focal Variable*			
Segregated Schooling			
≥ 50% of Grades 1-12	40.2		
1-49% of Grades 1-12	11.1		
No Segregated Schooling of Grades 1-12	48.5		
*Regional Schooling Effects*			
Majority of Schooling Occurred in the …			
Southern States	28.6		
Missouri	66.2		
Other States	5.2		
*Demographics, Socioeconomic Status, andHealth Status*			
Age (in years, 58–74)		65.8	4.5
Men (vs. women)	42.8		
Marital Status			
Divorced or Separated	27.7		
Widowed	18.5		
Never Married	10.0		
Married	44.8		
Educational Attainment			
Grade School	6.2		
High School	48.9		
College	44.8		
Employment Status			
Working for Pay	29.5		
Disabled	14.8		
Retired	55.7		
Annual Household Income < $25 K	42.9		
Self-Rated Health (1 = poor, …, 5 = excellent)		2.82	1.0
*Racial Consciousness, African AmericanCommunity Responsibility, and PerceivedDiscrimination*			
Constantly Thinks About One’s Race (1 = yes, 0 = no)	36.4		
Responsible to Move the African American Community Forward (1 = strongly disagree, …, 4 = strongly agree)		3.1	0.7
Perceived Discrimination (9 = lowest, …, 36 = highest)		24.7	4.5
*Resilience*			
Resilience (5 = lowest, …, 50 = highest)		40.6	6.4
*Outcome*^*1*^			
Sense of Control Scale (−16 = lowest, …, 16 = highest)		4.2	4.0
Systolic Blood Pressure (mm/hg; N = 511)		134.5	22.3
Diastolic Blood Pressure (mm/hg; N = 512)		76.8	13.0
Grip Strength (kg; N = 493)		32.0	11.9
Peak Expiratory Flow (ml; N = 442)		375.5	11.9
Gait Speed (0 = lowest, …, 5 = highest; N = 462)		2.4	1.2
Chair Stands (0 = lowest, …, 5 = highest; N = 462)		2.2	1.3
Balance Tests (0 = lowest, …, 5 = highest; N = 491)		3.2	1.5
Short Portable Physical Battery (0 = lowest, …, 12 = highest; N = 462)		7.9	3.1

^1^Sample size varies on the physical performance measures because (a) they could not be conducted for the 55 telephone and proxy interviews, (b) clinical condition rule-out screens were tripped (e.g., excessively high blood pressure for grip strength, or respiratory infections or conditions for peak expiratory flow), or (c) inadequate space in the home existed for the safe conduct of the tests (e.g., gait speed, or chair stands).

Distributions for the dependent variables were generally symmetrical, although modestly leptokurtic. The mean sense of control score was 4.2, consistent with national reports [7,8,15] and prior studies of disadvantaged and health challenged non-Hispanic Whites and African Americans in St. Louis and Indianapolis (Indiana) [55,56]. Average systolic (134.5 mm/hg) and diastolic (76.8 mm/hg) blood pressures were high. Grip strength (mean = 32.0 kg) and peak expiratory flow (mean = 375.5 ml) were about average. Mean gait speed, chair stands, and balance test categories were 2.4, 2.2, and 3.2, respectively. The average score on the SPPB was low (7.9), with 47.9% having scores (< 9) indicative of high risk for future adverse events.

### Multiple linear regression of the sense of control

Table 2 shows the unstandardized (*b*) regression coefficients obtained from modelling the sense of control. The unadjusted results (Model 1) indicate that the average sense of control score among participants with no segregated schooling was 3.73 (i.e., the intercept). While those who attended segregated schools for less than half of their 1^st^-to-12^th^ grade educations were not significantly different from those never attending segregated schools in terms of their sense of control scores, those attending segregated schools for half of more of their 1^st^-to-12^th^ grade educations did have significantly higher sense of control scores (4.72, *p* < .01), reflecting a standardized effect size of .25 (*b /* standard deviation). Introducing region of education in Model 2 increased the effect of segregated schooling for at least half of one’s 1^st^-to-12^th^ grade education (*b* = 1.239). The introduction of the traditional risk factors and potential confounders in Model 3, however, did not appreciably alter the effect of segregated schooling. Introducing the racial attitudes and beliefs in Model 4, and resilience in Model 5 also did not affect the effect of attending segregated schools for at least half of one’s 1^st^-to-12^th^ grade education (*b* = .886, p = .01, effect size = .22). The only other variables in Model 5 with statistically significant associations with the sense of control were traditional covariates: educational attainment, working for pay, income, and self-rated health. Higher (better) levels of the sense of control were associated with having more education, working for pay, having higher incomes, and enjoying better health.

**Table 2:** Unstandardized linear regression (*b*) coefficients obtained from predicting the sense of control (−16 to +16) at the 9-year follow-up in the African American Health cohort, N = 582

**Variable**	**Model 1**	**Model 2**	**Model 3**	**Model 4**	**Model 5**
*Focal Variable*					
Segregated Schooling					
≥ 50% of Grades 1-12	0.990**	1.239***	0.844**	0.922**	0.886**
1-49% of Grades 1-12	0.635	0.541	0.192	0.186	0.101
No Segregated Schooling in Grades 1-12 (reference category)					
*Regional Schooling Effects*					
Majority of Schooling Occurred in the …					
Southern States		−0.857*	−0.426	−0.488	−0.477
Missouri (reference category)					
Other States		2.093**	1.272	1.279	1.235
*Demographics, Socioeconomic Status, andHealth Status*					
Age (in years, 58–74)			0.020	0.010	0.008
Men (vs. women)			0.721*	0.722*	0.627
Marital Status					
Divorced or Separated			0.273	0.277	0.228
Widowed			0.324	0.298	0.277
Never Married			0.090	0.012	−0.004
Married (reference category)					
Educational Attainment (1 = grade school, 2 = high school, 3 = college)			0.917***	0.953***	0.939***
Employment Status					
Working for Pay			1.165**	1.188**	1.152**
Disabled			−1.133*	−1.011	−0.812
Retired (reference category)					
Annual Household Income < $25 K			−0.779*	−0.762*	−0.733*
Self-Rated Health (1 = poor, …, 5 = excellent)			0.546***	0.537**	0.519**
*Racial Consciousness, Community Responsibility,and Perceived Discrimination*					
Constantly Thinks About One’s Race (1 = yes, 0 = no)				−0.071	−0.090
Responsible to Move the African American Community Forward (1 = strongly disagree, …, 5 = strongly agree)				0.164	0.116
Perceived Discrimination (9 = highest, …, 36 = lowest)				−0.067	−0.068
*Resilience*					
Resilience (5 = lowest, …, 50 = highest)					0.043
Intercept	3.727***	3.776***	−1.443	−0.318	−1.640
R-squared	.014**	.040***	.165***	.171***	.175***

* = p < .05; ** = p < .01; *** = p < .001.

### Sensitivity analyses of the sense of control

Sensitivity analyses were conducted to better characterize the observed association between childhood school segregation and the overall sense of control score. Specifically, the two main components of the sense of control score—the sum of the responses to the four claiming control items, and the sum of the responses to the four denying control items—were examined using the same serial decomposition approach reflected in Table 2. Those results (not shown) indicate that the effect of childhood school segregation on the sense of control in later life is almost entirely derived from increased disagreement (and therefore greater sense of control) with the four items reflecting denying control. That is, the unstandardized regression coefficient (*b*) for having attended segregated schools for at least half of one’s 1^st^-to-12^th^ grade education obtained using the fifth (final) model was .867 (*p* = .003) for the denying control component scale, while its effect on the claiming control component scale was virtually nil (*b* = .020, *p* = .932).

### Multiple linear regression of physical performance

Table 3 shows the unstandardized (*b*) regression coefficients obtained from modelling the eight physical performance tests. The unadjusted results (Model 1) indicate that higher scores on the sense of control were associated with significantly lower systolic blood pressure, and with significantly better grip strength, peak expiratory flow, gait speed, chair stands, balance tests, and the SPPB (all *p* < .001). Adjustment for the two childhood school segregation measures in Model 2 did not appreciably alter these effects (all *p* < .001). Further adjustment for the remaining variables in Model 3 reduced the association to non-significance for gait speed, while the beneficial effects of the sense of control scores on the six other physical performance tests remained statistically significant.

**Table 3:** Unstandardized linear regression (*b*) coefficients for the sense of control obtained from predicting the physical performance measures at the 9-year follow-up in the African American Health cohort

	**N**	**Model 1**	**Model 2**	**Model 3**
**Physical Performance Measures**				
Systolic Blood Pressure (mm/hg)^1^	511	−0.826***	−0.826***	−0.730**
Diastolic Blood Pressure (mm/hg)^1^	512	−0.147	0.145	−0.207
Grip Strength (kg)^2^	493	0.654***	0.640***	0.285**
Peak Expiratory Flow (ml)^3^	442	7.774***	7.341***	2.743*
Gait Speed (0 to 5)^4^	462	0.056***	0.052***	0.019
Chair Stands (0 to 5)^4^	462	0.088***	0.087***	0.037**
Balance Tests (0 to 5)^4^	491	0.095***	0.091***	0.039**
Short Portable Physical Battery (0–12)^5^	462	0.237***	0.231***	0.105***

Note: Model 1 includes only the sense of control, Model 2 adds the two school segregation measures, and Model 3 adds the remaining covariates from Model 5 in Table 2.

^1^Average of three automated sphygmomanometer readings.

^2^Average of three trials with a hand held dynamometer.

^3^Average of three trials with a hand held flow meter.

^4^Scores of 0 reflect unable to perform, and 1–4 reflect national quartile thresholds.

^5^Scores are the sum of scores on the gait speed, chair stands, and balance tests.

* = p < .05; ** = p < .01; *** = p < .001.

### Mediation analyses

When the eight later life physical performance measures were regressed on the childhood segregation indicators, statistically significant beneficial associations were observed with peak expiratory flow, gait speed, the balance tests, and the SPPB. In addition, beneficial associations trending towards statistical significance were observed with grip strength and chair stands. Adjustment for the sense of control fully mediated the significant associations with gait speed and the SPPB, partially mediated the significant associations with peak expiratory flow and the balance tests, and eliminated the trend towards significance with grip strength and chair stands.

## Discussion

This study was driven by four hypotheses, all of which were logical extensions of Buck’s [24] work on the unintended consequences of desegregation leading to the erosion of the traditional “black community” school. The first hypothesis was that African Americans who attended segregated schools for their 1^st^-to-12^th^ grade education would have higher sense of control scores [13,54-57] (i.e., more claiming and less denying responsibility for both the good and bad things) in later life. The second hypothesis was that higher sense of control scores in later life would be related to better physical performance. The third hypothesis was that the effects of childhood school segregation on the sense of control in later life, and the effects of the sense of control on physical performance would hold even after adjustment for all of the traditional covariates and potential confounders, racial attitudes and beliefs, and resilience. The fourth hypothesis was that the beneficial association between childhood school segregation and physical performance in later life would be partially or fully mediated by the sense of control. Sense of control [13,54-57], school segregation, the traditional covariates and potential confounders [13,19,58], racial attitudes and beliefs [59,60], resilience [62], and physical performance [42,63-69] were all measured at the 2010 in-home follow-up for 582 of the 998 original AAH participants. Propensity score re-weighting methods [49-52] were used to adjust for potential attrition bias.

In terms of the first and third hypotheses, we found that the unadjusted effect of having attended segregated schools for at least half of one’s 1^st^-to-12^th^ grade education led to scores on the sense of control that were .990 points higher (*p* < .01, standardized effect size = .25) than those who did not attend segregated schools, indicating more claiming rather than denying control over both the good and bad outcomes in life. This significant association held even after adjusting for all of the traditional covariates and potential confounders [13,19,58], racial attitudes and beliefs [59,60], and resilience [62]. In the final model, African Americans who had received half or more of their 1^st^-to-12^th^ grade education in segregated schools had sense of control scores that were .886 points higher (*p* ≤ .01) than those who did not attend segregated schools, representing a standardized effect size of .22.

Sensitivity analyses that looked at the effects of childhood segregated schooling on each of the four-item component scores [13,55-57] of the sense of control—one reflecting claiming control and the other reflecting denying control—led to a better characterization of the observed association. Those results showed that the effect of childhood school segregation on the sense of control in later life was almost entirely derived from increased disagreement with the four items reflecting denying control (and therefore reflective of having a greater sense of control). Indeed, the unstandardized regression coefficient (*b*) for having attended segregated schools for at least half of one’s 1^st^-to-12^th^ grade education obtained from the fifth (final) model was .867 (*p* = .003) for the denying control component, which was almost as large as the effect on the overall sense control scale (where *b* = .886, *p* < .01). In contrast, there was no effect of segregated schooling on the claiming control component (*b* = .020, *p* = .932).

Recent work by Kraus et al. [70] provides insight into why denying control may have been most affected by childhood school segregation. Building on classic social science theory about why social class plays such a pervasive role in everyday life, they present a social cognitive theory as the underlying process by which social class creates clear cultural differences between the rich and the poor. The core of Kraus et al.’s argument is that:

"“…social class contexts elicit reliable social cognitive patterns among lower-class individuals—characterized by a contextual, externally-oriented cognitive and relational orientation to the world—and upper-class individuals—characterized by a solipsistic, individualistic cognitive and relational orientation to the world.” (p. 546)"

Although the contrast between the poor and the rich is not directly relevant here, the notion of how that contrast leads to the development of differential life orientations and outlooks is. Kraus et al. argue that:

"“Diminished resources and lower rank create contexts that constrain social outcomes for lower-class individuals and enhance contextualist tendencies—that is, a focus on external, uncontrollable social forces and other individuals who influence one's life outcomes.” (p. 546)"

African American children attending desegregated schools would have faced diminished educational resources and discrimination based on their ascribed racial status, and this may have led to their greater focus on and acceptance of external, uncontrollable forces in their lives. Thus, Kraus et al.’s explanation is entirely consistent with and provides the context for our findings. Indeed, if one were to substitute “childhood desegregated schooling” for “lower social class” and “childhood segregated schooling” for “upper social class” in their second and fifth hypotheses, our results would be exactly what they predicted.

Furthermore, our results are also consistent with, but clearly do *not* demonstrate Buck’s [24] main argument concerning the major unintended consequence of desegregation—the erosion of the traditional “black community” school. Buck argued that the erosion of the traditional “black community” school may have led to a gradual but significant cultural shift for subsequent generations of African Americans attending desegregated schools. Building on his argument, we hypothesized that this may specifically have led African Americans attending desegregated schools to be more likely to deny having any control over either the good or bad things happening in their lives. Buck also argued that over time, such cultural shifts may also have led to the emergence in the “black community” of the sentiment that African Americans with a stronger sense of responsibility for both the good and bad things occurring in their lives were “acting white.”

In terms of the second and third hypotheses, we found significant unadjusted associations between sense of control [13,55-57] scores and seven of the eight physical performance tests [38,63-69]. Higher scores on the sense of control were associated with significantly lower systolic blood pressure, and with significantly better grip strength, peak expiratory flow, gait speed, chair stands, balance tests, and the SPPB (all *p* < .001). Furthermore, adjustment for the two childhood school segregation measures, the traditional covariates and potential confounders [13,19,58], racial attitudes and beliefs [55,56], and resilience [62], did not appreciably alter the beneficial independent effects of the sense of control scores on systolic blood pressure, grip strength, peak expiratory flow, chair stands, balance tests, and the SPPB. While these effects are modest, they are important. Indeed, they indicate that a difference of just four points on the sense of control score would be associated with a one-point improvement on the SPPB, which would be enough to move the average AAH participant (mean = 7.9) above the cut-off for increased risk of future mobility impairment, poorer prognosis and recovery from newly diagnosed disease, and death [64-69].

In terms of the fourth hypothesis, we found statistically significant beneficial crude associations between childhood school segregation and physical performance for peak expiratory flow, gait speed, the balance tests, and the SPPB. The associations with peak expiratory flow and the balance tests were partially mediated by adjusting for the sense of control, and the associations with gait speed and the SPPB were fully mediated by adjusting for the sense of control. Thus, it would appear that childhood school segregation affected the sense of control in adult life, which resulted in better physical performance, suggesting that the sense of control plays an intervening variable role in the underlying causal sequence.

Although no other studies have focused on the association of segregated schooling and the sense of control [13,55-57] among older adults, recent rigorous studies of the effects that school segregation has on health outcomes for children and younger adults provide context and support for the results reported here [25-29]. In particular, data from the National Longitudinal Study of Adolescent Health (NLSAH) found that risky health behaviours such as smoking and drinking were lower for African American women in more segregated schools than for Non-Hispanic White women [34]. The two explanations given for this finding were that (a) because Non-Hispanic Whites had higher smoking and drinking rates, African American women in segregated schools may have been protected by the absence of cross-race friendships, and that (b) African American women in segregated schools may have engaged in other resiliency strategies [34]. Similarly protective functions associated with segregated schooling were found when the NLSAH was used to study depressive symptoms, which led the same authors to conclude that “attending predominantly-minority schools may buffer African American students from discrimination and increase their school attachment, which may reduce their risk of experiencing depressive and somatic symptoms” [35; p. 1873]. These explanations are consistent with the etiologic mechanism proposed by Buck [24] that drove our hypotheses extending his argument. It is important to note, however, that the NLSAH and the AAH reflect rather distinct cohorts of African Americans that lived through quite different historical and cultural periods in the United States, and are at rather different stages in the life course. Nonetheless, building on life course theory [71], both studies focus on the accumulative advantage [72] of segregated schooling for African Americans, at least in terms of less risky health behaviours and fewer depressive symptoms (in the NLSAH), and stronger sense of control and better physical performance (in the AAH).

It is important to note that this study is not without limitations. Three of these are most salient. The first involves the geographic generalizability of these associations beyond St. Louis, Missouri, and the extent to which these associations may be time-bound to the AAH cohort (born in 1936–1950). Therefore, further research on desegregation consequences from other communities and regions, as well as from other birth cohorts at different life stages is needed and should be encouraged. The second limitation is that the sense of control [13,55-57], school segregation, traditional covariates and potential confounders [13,19,58], racial attitudes and beliefs [59,60], resilience [62], and physical performance [42,63-69] were all measured at the 2010 in-home follow-up assessment. That makes ours a cross-sectional study in which we can only demonstrate associative relationships rather than causal effects. The third limitation is that our focal measures were retrospective self-perceptions of segregated schooling and the perceived extent of that exposure. As such, they are not objective, independent assessments of school segregation, and are subject to traditional retrospective reporting biases.

## Conclusions

In conclusion, we have shown that African Americans who attended segregated schools as children for at least half of their 1^st^-to-12^th^ grade educations were significantly more likely to claim rather than deny control over both the good and bad outcomes in their later lives, when compared to their counterparts who did not attend segregated schools as children. Moreover, higher sense of control scores in later life were associated with significantly lower systolic blood pressure, and with significantly better grip strength, peak expiratory flow, gait speed, chair stands, balance tests, and the SPPB in later life. While the etiologic mechanism proposed by Buck [24] could not be evaluated in these data, our results are consistent with the essence of his argument.

That said, *under no circumstances shouldour results be misconstruedas either evidence foror an argument thatdesegregation was bad socialpolicy*. Rather, our results suggest that (a) while desegregation may be a necessary condition for integration, it is not a sufficient condition, and (b) desegregation may have had an unfortunate, unintended consequence that led to lower sense of control in later life, with that lower sense of control associated with poorer physical performance. Assuming that our results are replicated in analyses from other studies, they have important policy and public health implications. To overcome these unfortunate side effects of desegregated schooling, greater emphasis may need to be placed on helping today’s desegregated schools (a) develop more and better same-race peer and authority role models for African American students, (b) emphasize that successful achievement avenues are not only possible but readily available, (c) minimize racial discrimination, and (d) promote cultural solidarity. Although achieving these goals will be difficult, the vibrancy of our society and civilization makes finding ways to accomplish them essential.

## Competing interests

The authors state that they have no competing interests.

## Authors’ contributions

FDW conceived of and planned the analyses reported here, performed all data analysis and reanalysis, and wrote and revised the manuscript. DKM conceived the overall plan for the AAH study, and contributed to the review of the analytic approach and revision of the manuscript. TKM contributed to data preparation, review of the analytic approach, and revision of the manuscript. JPM, MS, and EMA contributed to the review of the analytic approach and revision of the manuscript. All authors read and approved the final manuscript.

## Pre-publication history

The pre-publication history for this paper can be accessed here:

http://www.biomedcentral.com/1471-2458/12/827/prepub
